# Fezolinetant: A Potential Treatment for Moderate to Severe Vasomotor Symptoms of Menopause

**DOI:** 10.17925/EE.2023.19.2.13

**Published:** 2023-06-15

**Authors:** Barbara DePree

**Affiliations:** Holland Hospital Women's Specialty Care, Holland, MI, USA

**Keywords:** Fezolinetant, menopause, vasomotor symptoms, sleep, neurokinin-3 receptor antagonist, novel treatment option

## Abstract

The most common symptom of menopause is vasomotor symptoms (VMS), which occur in more than 80% of postmenopausal women. Furthermore, VMS are the manifestation of menopause for which women most commonly seek treatment, namely, to address their impacted quality of life, including sleep, and work-and non-work-related productivity. VMS vary in frequency, intensity and duration. Hormone therapy (HT) has been our most effective treatment for VMS and has been approved for this indication by the United States Food and Drug Administration (FDA). Despite being a safe and effective treatment option, many patients and providers are hesitant to consider HT. Moreover, HT is contraindicated for some women. While many over-the-counter and non-HT options are available, we lack data on the efficacy and safety of most of these. This has left a void for women. Fezolinetant was recently approved by the FDA for the treatment of moderate-to-severe VMS. So far, clinical trials have shown positive results in terms of safety and efficacy. Fezolinetant is a non-hormonal, neurokinin 3 receptor antagonist that works in the hypothalamus at the thermoregulatory centre. Blocking the non-hormonal neurokinin 3 receptor antagonist modulates hot flashes and night sweats. As early as 4 weeks from initiating fezolinetant, women experienced a statistically significant reduction of both severity and frequency of VMS per day, resulting in an improved quality of life.

During more than 30 years of clinical practice as a gynaecologist, I have observed that very few novel medications have had a significant impact in the field of women's health. There have been additions to selective oestrogen receptor modulators (SERMs), variations of hormone therapies (HTs), contraceptives and more. However, nearly all of these are modifications of existing treatment options. To date, none of them has been as potentially impactful as the introduction of fezolinetant and what it might mean for women seeking treatment for vasomotor symptoms (VMS) related to menopause.

## The impact of menopause

Menopause impacts 50% of the population, but this represents 100% of a gynaecologist's patients. In the USA, there were 63 million women over the age of 50 in 2021,^1^ with 6,000 women becoming menopausal every day.^2^ Given the current life expectancy, many women will spend up to 40% of their lives in postmenopause.^3^ Over the past two decades, we have moved beyond discussing menopause simply in terms of hot flashes or VMS to understanding the broader impact menopause has on women, including on productivity, both in the workplace and outside, and on overall quality of life, as well as its physical and emotional impact. Indeed, menopause is of no small consequence for many women.

Women's presence in the workforce has increased, notably in the past generation or two. UK data show that 45% of the workforce over 50 consists of women.^4^ Furthermore, the association between sleep disturbances and workplace productivity was assessed in a retrospective, longitudinal cohort study from the US Study of Women's Health Across the Nation (SWAN).^5^ The findings, published in 2021, showed that new-onset sleep problems in midlife women are associated with a significant increase in the risk of unemployment and about US$2 billion in lost productivity nationwide every year.^5^ The economics of menopause is emerging as an important factor in considering the healthcare management of these women. The mutual goal of provider and patient (and, in many cases, employers) is to allow them to remain active in their engagements, both paid and unpaid, as women are not only experienced and key employees but often also serve as caregivers for the older generation.

## Hormone therapy: challenges and alternatives

The media, celebrities, well-intentioned women who are going through menopause, tech gurus and even politicians are entering the conversation, seeking solutions and elevating the dialogue about the impact of menopause. Since their first report published in 2002, the Women's Health Initiative (WHI) has found that women and their healthcare providers are concerned about which treatments are considered safe and effective.^6^ Women have been avoiding HT and have been reluctant to return to using it,^6^ despite a growing body of evidence suggesting that HT is not only the most effective treatment for many menopausal symptoms, notably VMS, but it is also safe for most women.^7^ As fewer women have elected to use HT following the WHI report, and the emergence of data suggesting (misleadingly) the potential harm of using HT,^8^ providers have considered getting trained in HT management less of a priority.^9^ This has left a void for women seeking answers and solutions to bothersome menopausal symptoms, which has led to a boom in online sales and the marketing of numerous supplements claiming relief for symptoms but lacking evidence for their efficacy, let alone their safety.^10^ In addition, pharmaceutical companies have stepped in with their “bioidentical, custom-designed” options. In summary, it is a messy and challenging journey for women who are attempting to find solutions to menopausal symptoms on their own. They are desperate, and this situation is to their detriment. Women deserve to have the healthcare industry provide safe and effective therapies to address their menopausal symptoms.

## Vasomotor symptoms

Symptoms and conditions associated with menopause include VMS, sleep disturbances, genitourinary symptoms (e.g. vaginal dryness, urinary urgency, recurrent urinary tract infections, sexual dysfunction), skin changes, bone loss, joint pain, an increased risk of cardiovascular disease, diabetes and weight gain, changes in cognition, and depression. Of all the menopausal symptoms, VMS are the ones that are most likely to cause women to seek treatment.

Generally, women are dismissed when they complain about VMS; they are told that VMS are temporary, that the symptoms will get better over time, and that they should try to deal with it. However, VMS have been shown to have a significant negative effect on the quality of life of women in the USA, impacting their sleep, mood (e.g. depressive symptoms) and cognitive function.^11^ They frequently have an impact on work and relationships, as shared by my patients on a daily basis. Up to 80% of perimenopausal women in the SWAN study reported VMS in the 2 weeks prior to participating in the annual survey.^12^ Moreover, the prevalence of VMS in women in their late 50s has been reported to be between 30–50% when looking at data from 10 countries.^13^ In conclusion, many women are affected by VMS, often for many years and some for the remainder of their lifetime.

### Treatment options for vasomotor symptoms

Current treatment options for VMS generally fall into two categories: HT and non-HT. HT is the most common and effective treatment for VMS and has been approved by the United States Food and Drug Administration (FDA),^14^ not only for the treatment of VMS, but also for the treatment of genitourinary symptoms of menopause, the prevention of osteoporosis and for those women who experience premature hypo-oestrogenism. HT typically consists of oestrogen alone for women who no longer have a uterus and oestrogen plus progesterone/progestin for those with a uterus. It can have different formulations, doses and routes of administration. There is also the option of conjugated oestrogens/ bazedoxifene (Duavee™; Pfizer, New York, NY, USA), a tissue-selective oestrogen complex, which combines conjugated equine oestrogen with bazedoxifene, a SERM. The endometrial protection from the SERM in conjugated oestrogens/bazedoxifene does not require an added progestogen. Since the WHI raised safety concerns back in 2002, and given the multitude and nuance of the options available in HT, many providers are not well informed and/or they are reluctant to offer HT as a treatment option.

Non-HT includes selective serotonin reuptake inhibitors, clonidine, gabapentin, herbal products, nutritional supplements and lifestyle modifications. In 2013, the FDA approved paroxetine (Brisdelle; Noven Pharmaceuticals, Inc., Miami, FL, USA) 7.5 mg, a selective serotonin reuptake inhibitor, for treating VMS.^15^ To date, this has been the only non-HT approved by the FDA for VMS. The combination of tolerability, limited data on safety and efficacy, use of off-l abel medications, and the variable clinical response to non-HT has resulted in women being undertreated or untreated for their symptoms.

A recently published non-interventional, retrospective, observational cohort study evaluated the medical records of 1,016 women presenting with menopausal symptoms, including 91.2% who reported hot flashes, and found that nearly 40% of patients had no documentation of any prescription therapy offered, and 13% had no therapy documented at all, including prescriptions, non-prescription therapies and lifestyle interventions.^16^

### Fezolinetant

Fezolinetant has entered the market to help fill this void for women in menopause. Fezolinetant, a non-hormonal, neurokinin 3 receptor (NK3R) antagonist, is a novel treatment for VMS. The development of fezolinetant resulted from the physiology of the hot flash being further elucidated. The origin of the hot flash is in the thermoregulatory centre of the hypothalamus. This area of the brain is innervated by kisspeptin/ neurokinin B/dynorphin (KNDy) neurons. These KNDy neurons are stimulated by the neuropeptide neurokinin B, acting at the neurokinin 3 receptors, and this is inhibited by oestrogen. When oestrogen levels decline with the menopause transition, neurokinin 3 receptor-mediated activation is then unopposed in the absence of oestrogen. This, in turn, leads to the hypertrophy of the KNDy neurons and alters the activity of the thermoregulatory centre, resulting in hot flashes. Fezolinetant acts as a selective neurokinin 3 receptor antagonist. Blocking the NKB binding on the KNDy neuron leads to the restoration of normal thermoregulatory function and reduced episodes of heat dissipation that occur with a hot flash or night sweats.^17^

We now have the recently published data from the phase III randomized clinical tiral SKYLIGHT 1 trial assessing the efficacy and safety of fezolinetant for the treatment of moderate-to-severe VMS associated with menopause.^18^ This was a double-blinded, placebo-controlled, 12-week trial with a 40-week active treatment extension. The participants were women aged 40–65, experiencing a minimum average of 7 moderate-to-severe VMS per day. A total of 522 women were randomized to 12 weeks of once-daily placebo (n=175), fezolinetant 30 mg (n=173) or fezolinetant 45 mg (n=174). Those who completed 12 weeks were re-randomized to 30 or 45 mg of the medication for an additional 40 weeks. The coprimary efficacy endpoints were mean daily change from baseline to weeks 4 and 12 in VMS frequency and severity. A total of 355 women were analyzed for both efficacy and safety.^18^

The women in the study looked like the women we see in our office seeking care: 73% white and 14% African American, with a mean age of 54.4 and mean body mass index of 28.2; 13% were current smokers and mean time since the onset of VMS was 77 months. They all had 10–11 moderate-to-severe VMS per day at baseline.^18^

Both of the fezolinetant treatment doses (30 mg and 45 mg) met statistical significance in reducing VMS frequency and severity/day at weeks 4 and 12 compared with placebo. The reduction in hot flashes occurred within 1 week of initiation of the medication and was maintained for the 52 weeks of the trial.^18^ The placebo effect in hot flash research reports an average decrease of 25% in both frequency and intensity.^19^ There was a mean percentage reduction in VMS frequency of -56% for 30 mg and -61% for 45 mg (with a placebo response of -39.7%) at 12 weeks. This equates to a reduction in the number of hot flashes from a baseline of 10.7 to 4.5/d for 30 mg at week 12 and a reduction of 10.4 to 4.1/d for 45 mg at week 12 (with a placebo reduction from 10.5 to 6.9/d at week 12). This persistence of efficacy was maintained for the 52-week treatment duration.^18^

Reducing the frequency of VMS is important, but the reduction in severity may be more impactful to a woman's quality of life. The discomfort and disturbance of a severe hot flash impact when and how women engage in the world around them. Importantly, this study also evaluated how the clinical response to treatment translated into improved quality of life. Also evaluated, as secondary outcomes, in the 12-week period of treatments were the Menopause-specific Quality of Life (MENQOL) questionnaire and Patient-Reported Outcomes Measurement Information System Sleep Disturbance—Short Form 8b (PROMIS 8D SF 8b). The MENQOL questionnaire is a 29-i tem measure of 4 domains: vasomotor, psychosocial, physical, and sexual.^20^ PROMIS 8D SF 8b is an assessment of 8 aspects of sleep disturbance. The reduction in VMS frequency and severity resulted in meaningful improvements in quality of life reported by the MENQOL questionnaire, with both doses significantly improving quality of life as early as week 4. The improvements in sleep disturbance for fezolinetant 30 mg and 45 mg versus placebo did not reach statistical significance in the phase III clinical trial SKYLIGHT 1 (ClinicalTrials.gov identifier: NCT04003155)^18^ as is did in the clinical trial SKYLIGHT 2 (ClinicalTrials.gov identifier: NCT04003142).^21^

While hot flashes and night sweats are the most common symptoms experienced by women going through menopause, it is often the sleep disruption that accompanies the night sweats that causes women to seek treatment. It has been reported that nearly half of postmenopausal women complain about sleep disruption, with VMS often being the inciting cause.^22^ Lack of good quality sleep is a life stressor for many women. Women with VMS and sleep complaints are also more likely to suffer from symptoms of depression.^23^

While it is great to have a new and effective treatment option for VMS, our patients are also very interested in safety. The WHI outcomes and the media communication of the original findings caused not only panic in women who felt they had been misled about the safety of their HT but also, I think, some lasting mistrust between patients and providers and between patients and the pharmaceutical industry. While this is a completely new class of medication with no hormonal activity, patients are likely to be somewhat sceptical of fezolinetant and deserve to have safety be a central feature of this medication for the treatment of VMS.

The randomized, double-blind, 52-week, phase III safety study SKYLIGHT 4 of fezolinetant 30 mg and 45 mg was recently published (ClinicalTrials.gov identifier: NCT04003389).^24^ The primary endpoints were treatment-emergent adverse events (TEAEs), the percentage of women with endometrial hyperplasia and endometrial malignancy. A total of 1,830 postmenopausal women seeking treatment for VMS were randomized equally to receive placebo, fezolinetant 30 mg or fezolinetant 45 mg. There was one occurrence of endometrial hyperplasia in the fezolinetant 45 mg group and none in the placebo or the fezolinetant 30 mg group. There was one case of endometrial cancer in the fezolinetant 30 mg group and none in the placebo or fezolinetant 45 mg group. Uterine bleeding occurred in 4.9% of patients in the placebo group, 3.3% of those in the fezolinetant 30 mg group, and 3.1% of those in the fezolinetant 45 mg group. These findings suggest that using fezolinetant does not cause any safety concerns for the endometrium.^24^

TEAEs occurred in 64.1% of the placebo group, 67.9% of the fezolinetant 30 mg group, and 63.9% of the fezolinetant 45 mg group.^24^ These adverse events were generally mild to moderate, with the most common being headache and coronavirus disease 2019; no significant differences were seen between the three groups. Serous TEAEs had a low incidence across all three groups: 2.3% for placebo, 3.3% in 30 mg, and 3.8% in 45 mg. In the study, the only death occurred in the fezolinetant 30 mg group and was not considered related to the medication. Discontinuation of the medication related to the TEAEs was low and similar across the three groups: 4.3% in the placebo group, 5.6% in the fezolinetant 30 mg group and 4.6% in the fezolinetant 45 mg.^24^

## Future considerations

It is a relief to know that more women will now have access to safe and effective therapy for VMS. The fact that it is not HT will lower the bar to access for both patients and many providers. Despite being over 20 years since the initial publication of the WHI findings suggesting risks with HT, the plethora of data supporting the safe use of HT and having a new generation of women entering menopause, there is still lingering reluctance to adopt HT. Women are reluctant to consider HT for the treatment of their menopausal symptoms, and providers are reluctant to prescribe and manage HT. In this context, fezolinetant offers a safe and effective alternative to HT.

My concern with the introduction of this medication is that it might deny HT access to women who would most benefit from it, especially women who are undergoing premature menopause. We now understand the importance of oestrogen exposure until the natural age of menopause (the average age is 51 in the USA) to maintain and promote bone, cardiovascular and brain health.^25^ For women with *BRCA1* and *BRCA2* mutations, who have had risk-reduction surgeries and premature menopause as a result, the use of HT is beneficial for reducing chronic disease.^26^ Fezolinetant would not provide that same protection for these younger women.

Many women complain about multiple bothersome symptoms when they are going through menopause, including (but not exclusively) joint pain, mood disturbances, painful intercourse, urinary frequency, weight gain, brain fog and skin changes. For these women, who have multiple symptoms associated with menopause, addressing VMS with fezolinetant may not adequately address their concerns. However, fezolinetant is likely going to be a very good option for women whose primary issue is VMS.

While most women and practitioners have viewed VMS as simply a quality of life issue, there is growing evidence linking VMS with cardiovascular disease and poor bone health,^27^ two conditions that have significant morbidity and mortality for postmenopausal women. If reducing VMS with fezolinetant could ameliorate these symptoms, this treatment option could improve health outcomes. I look forward to more research and to continued understanding of this.

## Conclusions

How intertwined are VMS, sleep and mood? Will addressing VMS and, in turn, sleep be a solution for improving mood? I do not currently have answers to these questions, but I am looking forward to our patients and additional research teaching us more in the coming years with the introduction of fezolinetant.

I intend to discuss this product with my patients. When I speak about the recent approval of a new menopausal VMS treatment for women to my patients, they seem grateful to know that there will be additional options and that science is forging ahead with investigating and finding solutions to address this universal process: menopause. Thirty to forty years is a long portion of a woman's lifespan; consequently, we should be actively seeking answers to address the impact of reduced hormone production that accompanies menopause.
